# Plasma after both SARS-CoV-2 boosted vaccination and COVID-19 potently neutralizes BQ.1.1 and XBB.1

**DOI:** 10.1099/jgv.0.001854

**Published:** 2023-05-11

**Authors:** David J. Sullivan, Massimo Franchini, Jonathon W. Senefeld, Michael J. Joyner, Arturo Casadevall, Daniele Focosi

**Affiliations:** ^1^​ Johns Hopkins Bloomberg School of Public Health and School of Medicine, Baltimore, MD 21218, USA; ^2^​ Division of Transfusion Medicine, Carlo Poma Hospital, 46100 Mantua, Italy; ^3^​ Department of Anesthesiology & Perioperative Medicine, Mayo Clinic, Rochester, MN 55902, USA; ^4^​ North-Western Tuscany Blood Bank, Pisa University Hospital, 56124 Pisa, Italy

**Keywords:** convalescent plasma, SARS-CoV-2, COVID-19, BQ.1.1, XBB, BF.7: virus neutralization

## Abstract

Recent 2022 SARS-CoV-2 Omicron variants, have acquired resistance to most neutralizing anti-Spike monoclonal antibodies authorized, and the BQ.1.* sublineages are notably resistant to all authorized monoclonal antibodies. Polyclonal antibodies from individuals both vaccinated and recently recovered from Omicron COVID-19 (VaxCCP) could retain new Omicron neutralizing activity. Here we reviewed BQ.1.* virus neutralization data from 920 individual patient samples from 43 separate cohorts defined by boosted vaccinations (Vax) with or without recent Omicron COVID-19, as well as infection without vaccination (CCP) to determine level of BQ.1.* neutralizing antibodies and percent of plasma samples with neutralizing activity. More than 90 % of the plasma samples from individuals in the recently (within 6 months) boosted VaxCCP study cohorts neutralized BQ.1.1, and BF.7 with 100 % neutralization of WA-1, BA.4/5, BA.4.6 and BA.2.75. The geometric mean of the geometric mean 50 % neutralizing titres (GM (GMT_50_) were 314, 78 and 204 for BQ.1.1, XBB.1 and BF.7, respectively. Compared to VaxCCP, plasma sampled from COVID-19 naïve subjects who also recently (within 6 months) received at least a third vaccine dose had about half of the GM (GMT_50_) for all viral variants. Boosted VaxCCP characterized by either recent vaccine dose or infection event within 6 months represents a robust, variant-resilient, neutralizing antibody source against the new Omicron BQ.1.1, XBB.1 and BF.7 variants.

## Impact Statement

Recently collected polyclonal plasma from individuals who are both vaccinated and have recovered COVID-19: (i) neutralizes mismatched future variants including BQ.1.1 and (ii) neutralizes monoclonal resistant variants of SARS-CoV-2.

## Introduction

In immunocompromised (IC) patients both passive immunotherapies and small molecule antivirals are often necessary to treat COVID-19 or eliminate persistently high SARS-CoV-2 viral load. Chronic, persistent viral loads increase both transmission and mutation risk, and prevent administration of the required immunosuppressive/antineoplastic therapies [1]. Small molecule antivirals have not been formally validated for IC patients, who often have contraindications, and the convergent evolution of the Omicron variant of concern (VOC) has led to inefficacy of all the anti-Spike monoclonal antibodies (mAbs) authorized so far for both treatment or prevention, e.g. in the highly prevalent BQ.1.* sublineages [2]. The other rapidly spreading XBB.* and BF.7 sublineages are also highly resistant to anti-Spike mAbs [3]. Polyclonal plasma from individuals who are both vaccinated and have recovered from COVID-19 (VaxCCP) has more than ten times the antibody levels capable of neutralizing pre-Omicron variants as well as Omicron variants BA.1 through BA.4/5 [4, 5]. Polyclonal COVID-19 convalescent plasma (CCP) has thousands of distinct antibody specificities of different isotypes targeting SARS-CoV-2, including hundreds capable of SARS-CoV-2 higher affinity virus neutralization by interference with angiotensin converting enzyme 2 [6]. Approximately 800 diverse epitopes have been mapped to the SARS-CoV-2 genome with more than ten implicated important to neutralization [7]. High-titre pre-Omicron CCP contains Omicron neutralizing activity despite being collected before variant appearance [4, 5].

Given that CCP remains a recommended therapy for IC [1, 8, 9], we systematically reviewed recent primary research for neutralization results against BQ.1.1 and to a limited extent XBB.1 by plasma collected from vaccinated subjects with or without COVID-19 or after recent Omicron infection alone.

## Methods

**Fig. 1.**
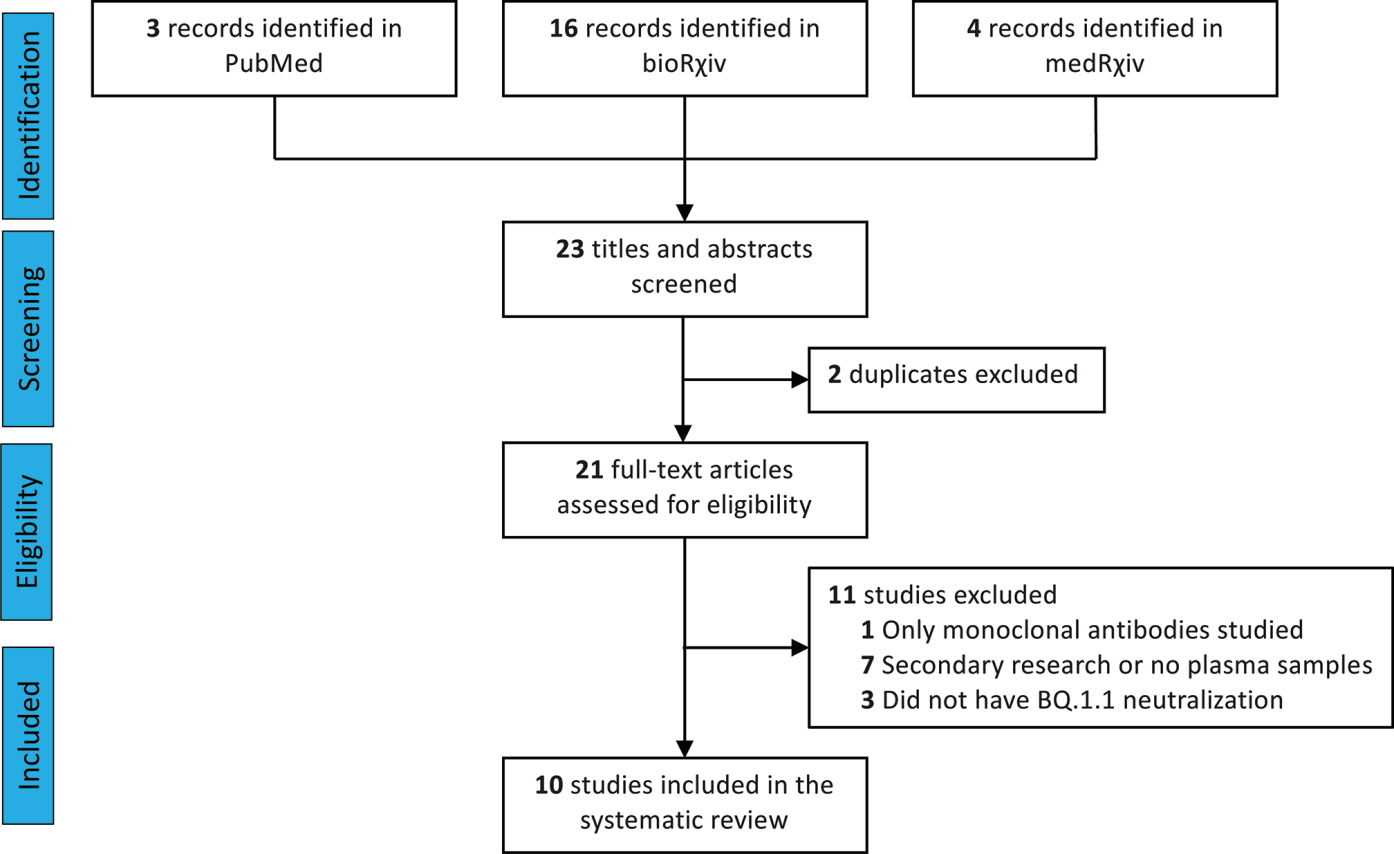
PRISMA flowchart for the current study. Number of records identified from various sources, excluded by manual screening, found eligible and included according to subgroup analyses.

On 19 November 2022 we initially searched PubMed, medRxiv and bioRxiv for manuscripts reporting BQ.1.1 neutralization, using English language as a restriction. Search of bioRxiv with same keywords now yields 17 records of which only ten contained plasma viral neutralization data. Search of medRxiv produced three records which did not have BQ.1.1 neutralizations. PubMed retrieved three entries using (‘BQ.1.1’) and (‘neutralization’), one of which was focused on anti-Spike mAb alone [2] and the other two were duplicates from bioRxiv [10, 11]. Articles underwent evaluation for data extraction by two assessors (D.S. and D.F.) with disagreements resolved by third assessor (A.C.). Articles lacking plasma BQ.1.1 virus neutralizations were excluded. The process of study selection is represented in the PRISMA flow diagram (Fig. 1).

The type of viral assay (live or pseudovirus), time interval to blood sample, GMT_50_, minimum and maximum neutralizing 50 % dilutional titre for WA-1 (pre-Alpha wild-type) and Omicron sublineages BQ.1.1, BA.4/5, BA.4.6, BA.2.75, XBB.1 and BF.7 and number out of total that neutralized Omicron were abstracted from study text, graphs and tables. Two studies (Wang [12] and Qu [10]) reported BQ.1 and those were separate cohorts in addition to BQ.1.1. Prism v. 9.4 (GraphPad Software, San Diego, CA, USA) was used for data analysis. While all manuscripts included neutralization data against WA-1, BQ.1.1, BA.4/5 and BA.2.75, only a subset of manuscripts included neutralization data for BA.4.6, XBB.1 and BF.7 which were assembled for relevance to present circulating variants. Historic early Omicron partial neutralization data on variants like BA.1 or BA.2 were excluded because of the full set data with BA.4/5 and BA.2.75.

We performed a geometric mean of the reported study cohort reported GMT_50_. Statistical significance between log_10_ transformed GMT_50_ was investigated using Tukey’s test. The multiple comparison test was a two-way ANOVA with Alpha 0.05 on log transformed GMT_50_. The log normal test was performed on WA-1, BQ.1.1, BA.4/5, BA.4.6, XBB.1 and BF.7 virus GMT_50_.

The percent of virus neutralizations used the individual study threshold in Table S1 (available in the online version of this article), to report the total number that were neutralized or not. In contrast the GM (GMT_50_) is a geomean of 5–23 patient cohorts extracted from the ten published studies and not from raw data that was not available on each sample dilutional titre virus neutralization. Two studies [13, 14] reported the median titre rather than the GMT_50_. Compiled data abstracted from the published studies is available in the supplementary dataset.

## Results

Ten articles were included (Fig. 1) which contained virus neutralizations with WA-1, BQ.1.1, BA.4/5, BA.4.6, XBB.1 and BF.7, assessed with either live authentic SARS-CoV-2 or SARS-CoV-2 pseudovirus neutralization assays and represented data from 920 patients (Table S1). Qu *et al.* in the USA reported on Spring and Summer 2022 with BA.1 and BA.4/5 infections in two sampled cohorts with predominantly unvaccinated individuals, as well as a third cohort of healthcare workers after a single monovalent booster vaccination in the Fall of 2021 [10] (Table 1). Zou *et al.* in the USA in the Summer and Fall of 2022 sampled individuals who had already received three mRNA BNT162b2 vaccinations with or without previous COVID-19, both before and about 4 weeks after a fourth monovalent or bivalent vaccine booster vaccination [15]. Miller *et al.* also in the USA sampled both before the third vaccination dose and about 4 weeks after monovalent mRNA vaccination in the Fall of 2021, as well as with the fourth vaccine dose in the Summer or Fall of 2022, with either monovalent or bivalent booster vaccinations in Fall of 2022 in those with no documented COVID-19 [13]. Cao *et al.* in China investigated BQ.1.1 neutralizations from plasma of four cohorts after three doses of CoronaVac (Fall 2021) without COVID-19 or 2–12 weeks after BA.1, BA.2 and BA.5 infection [3]. Planas *et al.* in France evaluated GMT_50_ in plasma from individuals both 4 and 16 weeks after a third monovalent mRNA vaccine dose in the Fall of 2021 as well as 12 and 32 weeks after vaccine breakthrough BA.1/2 or BA.5 infection [14]. Davis *et al.* in the USA sampled after the third mRNA vaccine monovalent dose in the Fall of 2021 and also after either a fourth monovalent mRNA dose or a bivalent (wild-type +BA0.4/5) vaccine dose in the Summer and Fall of 2022 [11]. Kurhade *et al.* in the USA also compared GMT_50_ after the fourth monovalent vaccine dose or three mRNA doses with the fourth the bivalent dose without COVID-19 and also after bivalent boost with recent COVID-19 [16]. Wang *et al.* in the USA compared GMT_50_ after three vaccine doses, the fourth monovalent vaccine dose or three mRNA doses with the fourth the bivalent dose without COVID-19, and also after 2–3 vaccine doses and recent BA.2 breakthrough infection or 3–4 mRNA vaccine doses and recent BA.4/5 breakthrough infection [12]. Ito *et al.* in Japan compared breakthrough infections after BA.2 and BA.5 after 2–3 doses of mRNA vaccines in the Spring and Summer of 2022 [17]. Akerman *et al.* in Australia characterized neutralizing antibodies in four groups 1) sampling one to 3 months after three doses of mRNA vaccines with an Omicron infection in 2022; 2) sampling 3 months after four doses of mRNA vaccine; 3) sampling 6 months after three doses of mRNA vaccine and 4) sampling 3–6 months after last vaccine in a larger cohort who had the original WA-1 infection in early 2020 as well as three more doses of mRNA vaccine [18].

**Table 1.** Synopsis of included studies, reporting plasma sources, epoch of sampling, region, time since vaccination/infection to plasma sampling, and sample size. The cohorts were split into four groups–1) boosted vaccinations and recent COVID-19 (VaxCCP), 2) boosted vaccines only without documented COVID-19 (Vax only) and 3) infection alone (CCP) or pre-boosted sampling before third or fourth vaccine dose (preVax)

Study	Vaccine and COVID-19 history at sample time	Group	Time period of plasma sampling	Geography	Sampling time mean or median (min-max)	Sample no.
Cao [3]	3×CorVac+BA0.1 inf	VaxCCP	Spring 2022	China	5–7 weeks post-hosp admit (42 weeks avg)	50
Cao [3]	3×CorVac+BA0.2 inf	VaxCCP	Summer 2022	China	3–11 weeks post-hosp admit (8 weeks mean)	39
Cao [3]	3×CorVac+BA0.5 inf	VaxCCP	Summer/Fall 2022	China	2–11 weeks (mean 5 weeks)	36
Zou [15]	4×BNT162b2+BTI	VaxCCP	Summer/Fall 2022	USA	4 weeks post-dose	20
Planas [14]	mRNAvac×3 +BA0.1/2 inf	VaxCCP	Spring/Fall 2022	France	32 weeks post-BTI BA.1/2	13
Wang [12]	2–3×mRNAvac+BA0.2 BTI	VaxCCP	Spring/Fall 2022	USA	2–23 weeks (mean 6 wk (3over 90 days))	14
Wang [12]	3–4×mRNAvac+BA0.4/5 BTI	VaxCCP	Summer/Fall 2022	USA	2–8 weeks (mean 4 weeks)	20
Kurhade [16]	3×mRNAvac+bivalent+BTI	VaxCCP	Fall 2022	USA	4 weeks post-with infection history	23
Ito [17]	2–3×mRNAvac+BA0.2 BTI	VaxCCP	Spring 2022	Japan	2–8 weeks	14
Ito [17]	2–3×mRNAvac+BA0.5 BTI	VaxCCP	Summer 2022	Japan	2–3 weeks	20
Zou [15]	3×BNT162b2+bivalent+BTI	VaxCCP	Summer/Fall 2022	USA	4 weeks post-dose	19
Akerman [18]	3×mRNA+bivalent	VaxCCP	Fall 2022	Australia	4–12 weeks post-BTI	29
Planas [14]	3×mRNAvac+BA0.1/2 inf	VaxCCP	Spring/Fall 2022	France	12 weeks post-BTI BA.1/2	16
Planas [14]	3×mRNAvac+BA0.5 inf	VaxCCP	Fall 2022	France	8 weeks post-BTI BA.5	15
Davis [11]	3×mRNAvac	Vax only	Fall 2021	USA	1–4 weeks post-boost	12
Kurhade [16]	4×mRNAvac	Vax only	Summer 2022	USA	4–12 weeks	25
Cao [3]	3×CorVac	Vax only	Fall 2021	China	4 weeks	40
Zou [15]	4×BNT162b2	Vax only	Summer/Fall 2022	USA	4 weeks post-dose	20
Planas [14]	3×mRNAvac	Vax only	Winter 2021/2022	France	16 weeks post-3rd dose	10
Wang [12]	3×mRNAvac	Vax only	Fall 2021	USA	2–12 weeks (mean 5 weeks)	14
Wang [12]	3×mRNAvac+monovalent	Vax only	Summer/Fall 2022	USA	3–4 weeks	19
Wang [12]	3×mRNAvac+bivalent	Vax only	Summer/Fall 2022	USA	3–4 weeks	21
Davis [11]	3×mRNAvac+monovalent	Vax only	Summer/Fall 2022	USA	10–15 weeks post-boost	12
Akerman [18]	4×mRNA	Vax only	Fall 2022	Australia	12 weeks	23
Akerman [18]	3×mRNAvac after 2020 WA-1	Vax only	Summer/Fall 2022	Australia	3–6 months	47
Kurhade [16]	3×mRNAvac+bivalent	Vax only	Fall 2022	USA	4 weeks post	29
Davis [11]	3×mRNAvac+bivalent	Vax only	Summer/Fall 2022	USA	2–6 weeks post-booster (8 no vacc. 10 no infection)	12
Qu [10]	3×mRNAvac	Vax only	Fall 2021	USA	2–13 weeks	15
Zou [15]	3×BNT162b2+bivalent	Vax only	Summer/Fall 2022	USA	4 week post-dose	18
Planas [14]	3×mRNAvac	Vax only	Fall/Winter 2021	France	4 weeks post-3rd dose	18
Miller [13]	3×BNT162b2	Vax only	Fall 2021	USA	2–4 weeks	16
Miller [13]	3×mRNA+monovalent	Vax only	Spring/Fall 2022	USA	2–9 weeks	18
Miller [13]	3×mRNA+bivalent	Vax only	Fall 2022	USA	2–3 weeks	15
Qu [10]	BA.4/5 inf (17-unvac)	CCP	Summer 2022	USA	not stated	20
Qu [10]	Hosp BA.1 (6-unvac;5-2×mRNAvac)	CCP	Spring 2022	USA	1 week post-hospitalization	15
Zou [15]	3×BNT162b2+BTI	preVax with BNT162b	Summer/Fall 2022	USA	preboost with BNT162b (6–11 months post-last dose)	20
Zou [15]	3×BNT162b2+BTI	preVax with bivalent	Summer/Fall 2022	USA	preboost with bivalent (6–11 months post-last dose)	19
Zou [15]	3×BNT162b2	preVax with bivalent	Summer/Fall 2022	USA	preboost with bivalent (6–11 months post-last dose)	18
Zou [15]	3×BNT162b2	preVax with BNT162b	Summer/Fall 2022	USA	preboost with BNT162b (6–11 months post-last dose)	20
Akerman [18]	3×mRNA	preVax	Fall 2022	Australia	preboost (6 months)	47
Miller [13]	2×BNT162b2	preVax with BNT162b	Fall 2021	USA	preboost (6–11 months post-last dose)	16
Miller [13]	3×mRNA	preVax with bivalent	Fall 2022	USA	preboost with bivalent (6–11 months post-last dose)	15
Miller [13]	3×mRNA	preVax with monovalent	Spring/Fall 2022	USA	preboost with monovalent (6–11 months post-last dose)	18

BTI, Breakthrough Infection; Vax, Vaccination; VaxCCP, Hybrid vaccination and recent COVID-19.

**Fig. 2.**
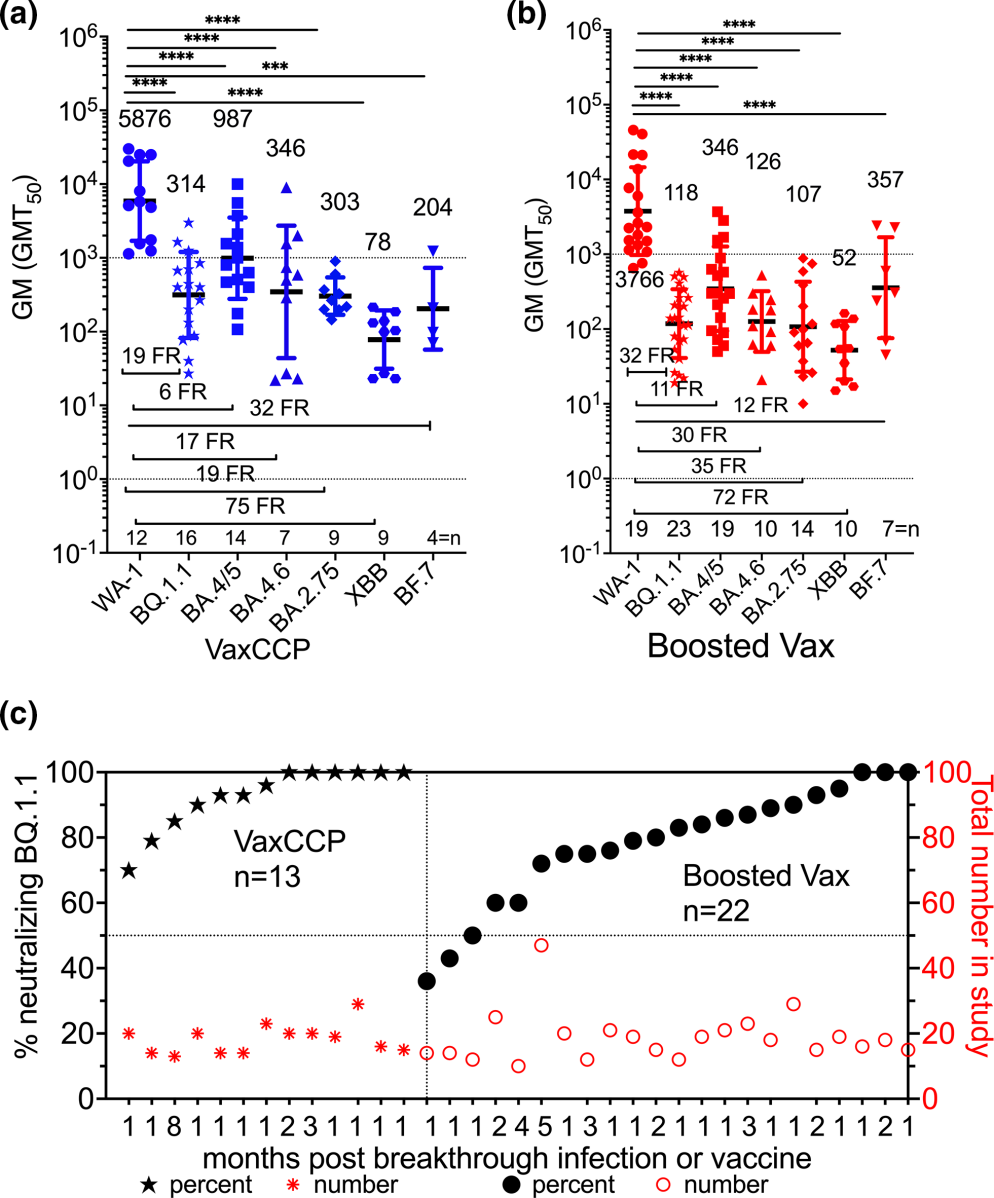
Neutralizing GM (GMT_50_) against WA-1, BQ.1.1, BA.4/5, BA.4.6, BA.2.75, XBB, BF.7. (**a**) VaxCCP and (b) boosted Vax plasma without COVID-19. Number above the study points are geometric mean of the individual studies GMT_50_ with standard deviation for error bars. The fold reduction (FR) are below data study points, and number of individual studies for each variant virus are next to and above x-axis. Geomeans statistically significant in difference by multiple comparison in Tukey’s test are indicated. (**c**) The percent of total samples within each study cohort which neutralized Omicron BQ.1.1 graphed in increasing percentages on left y-axis with the total number of individual samples tested on the right y-axis. The x-axis numbers are the months after either recent COVID-19 or vaccination that sample was collected. The percent neutralizations after CoronaVac and Omicron COVID-19 (VaxCCP) in three cohorts and Vax in one cohort in the paper by Cao *et al.* could not be retrieved from the manuscript.

These diverse cohorts were assembled into four groups, 1) plasma after both 2–4 vaccine doses and COVID-19 (VaxCCP); 2) plasma from subjects after administration of 3–4 vaccine doses (i.e. boosted), but either self-reported as COVID-19-naïve or anti-nucleocapsid negative (Vax); and 3) Omicron infection without vaccination (CCP) as well as participants sampled 6 to 11 months after previous vaccine dose and before the booster vaccination (preVax). Boosted VaxCCP neutralized BQ.1.1, XBB.1 and BF.7 with approximately three times the dilutional potency of the Vax-only or 2–6 times preVax groups for all viral variants (Table 2 and Fig. 2). Importantly, while there was a 19-fold reduction in neutralization by boosted VaxCCP against BQ.1.1 compared to WA-1, more than 90 % of the boosted VaxCCP samples neutralized BQ.1.1 as well as XBB.1 and BF.7 (Table 2 and Fig. 2c). The neutralization thresholds varied in each study with the live virus assays 20–30 and pseudovirus 20–150 (Table S1). Three cohorts within the boosted VaxCCP group were below at 90 % neutralization with one sampled late, 8 months after BA.1/2 breakthrough infection [14] and the other two from a single study after BA.2 and BA.5 (Tables S2 and S3). Except for the GM (GMT_50_) against XBB.1 at 78, the other viral variant neutralizations were in the same range as pre-Alpha CCP neutralizing WA-1 (i.e. 311) [4]. By comparison the large randomized clinical trial which effectively reduced outpatient COVID-19 progression to hospitalizations had a GMT_50_ of 60 for WA-1 with pre-Alpha CCP [19]. Boosted Vax at 3–4 doses without COVID-19, showed GM (GMT_50_) of 118 for BQ.1.1, with only 6 of 23 cohorts over 90 % neutralizations, for 79 % overall (i.e. 326 of 414 individuals). Four separate studies [10, 11, 13, 16] characterized BQ.1.1 virus neutralizations with plasma after the new bivalent (wild-type +BA0.4/5) mRNA vaccine booster in the Fall of 2022, with 88 % (103 of 117 samples) neutralization activity within 4 weeks of bivalent booster (Table S3).

**Table 2.** GM (GMT_50_) of plasma from three different sources against recent Omicron sublineages

Neutralization virus	WA-1	BQ.1.1	BA.4/5	BA.4.6	BA.2.75	XBB.1	BF.7
**VaxCCP (study cohorts**)	12	16	14	7	9	9	4
GM (GMT_50_)	5876∗	314	987	346	303	78	204
Fold reduction from WA-1	ref	19	6	17	19	75	32
Samples tested	294	237†	328	135	231	125	148
Samples neutralizing	285	221	326	135	230	111	146
Percent neutralizing	97	93	99	100	100	89	99
**Boosted Vax (study cohorts**)	19	23	19	10	14	10	7
GM (GMT_50_)	3766	118	346	126	107	52	357
Fold reduction from WA-1	ref	32	11	30	35	72	12
Samples tested	384	414†	384	206	261	217	158
Samples neutralizing	383	326	363	191	231	125	149
Percent neutralizing	100	79	95	93	89	58	94
**CCP only or preVax (study cohorts**)	10	12	10	7	9	5	5
GM (GMT_50_)	870	47	96	103	57	23	110
Fold reduction from WA-1	ref	19	9	8	15	38	9
Samples tested	184	220	184	136	162	101	84
Samples neutralizing	182	139	144	104	104	50	63
Percent neutralizing	99	63	78	76	64	50	75

* Pre-Alpha CCP from 27 different studies had a GM (GMT_50_) of 311 from 707 samples with 315 or 45 % neutralizing omicron BA.1 [4].

† Percent neutralizations after CoronaVac and Omicron COVID-19 (VaxCCP) and Vax in the paper by Cao *et al.* could not be retrieved from the manuscript. So 237 samples from the six other cohorts were used for percent neutralization.

CCP, COVID-19 Convalescent Plasma; GM(GMT50), Geometric mean of Geometric mean titres at 50% inhibiton; Vax, Vaccination; VaxCCP, Hybrid vaccination and recent COVID-19.

**Fig. 3.**
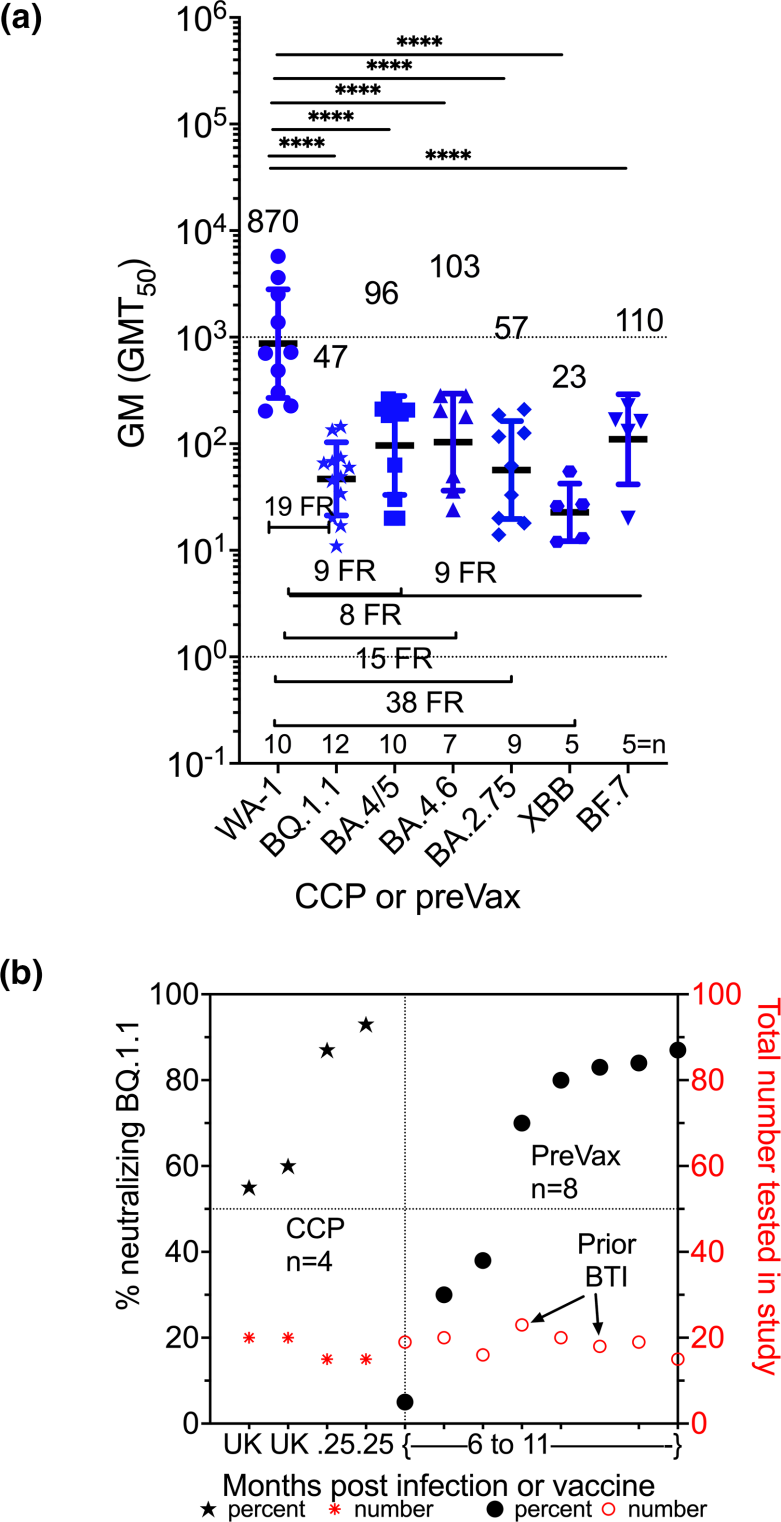
Geometric mean neutralizing titres (GMT_50_) against WA-1, BQ.1.1, BA.4/5, BA.4.6, BA.2.75, XBB, BF.7. (a) plasma Omicron CCP alone or preVax-6 to 11 months after last vaccine dose sampled in 2021 or 2022. Number above the study points are geometric mean of the individual studies GMT_50_ with standard deviation for error bars. The fold reduction (FR) are below data study points, and number of individual studies for each variant virus are next to and above x-axis. GM (GMT_50_) statistically significant in difference by multiple comparison in Tukey’s test are indicated. (**b**). The percent of total samples within each study cohort which neutralized Omicron BQ.1.1 graphed in increasing percentages on left y-axis with the total number of individual samples tested on the right y-axis. The x-axis numbers are the months after either recent COVID-19 or vaccination that sample was collected. UK is unknown and 6–11 means more than 6 months. Prior BTI is a previous breakthough infection months before the preVax sample.

Many studies performed virus neutralizations on samples drawn before the third or fourth vaccine dose which were 6 to 11 months after last vaccine dose or preVax. The GM (GMT_50_)’s for BQ.1.1 and BA.2.75 for preVax were about six times reduced compared to VaxCCP even though the fold reductions were similar (Fig. 3, Table 2). In agreement with lower GM (GMT_50_) for neutralizations was the low percent neutralizing BQ.1.1 (63 %), XBB.1 (50 %), and BF.7 (75 %) at 6 to 11 months after vaccination (Fig. 3, Tables 2 and S3).

**Fig. 4.**
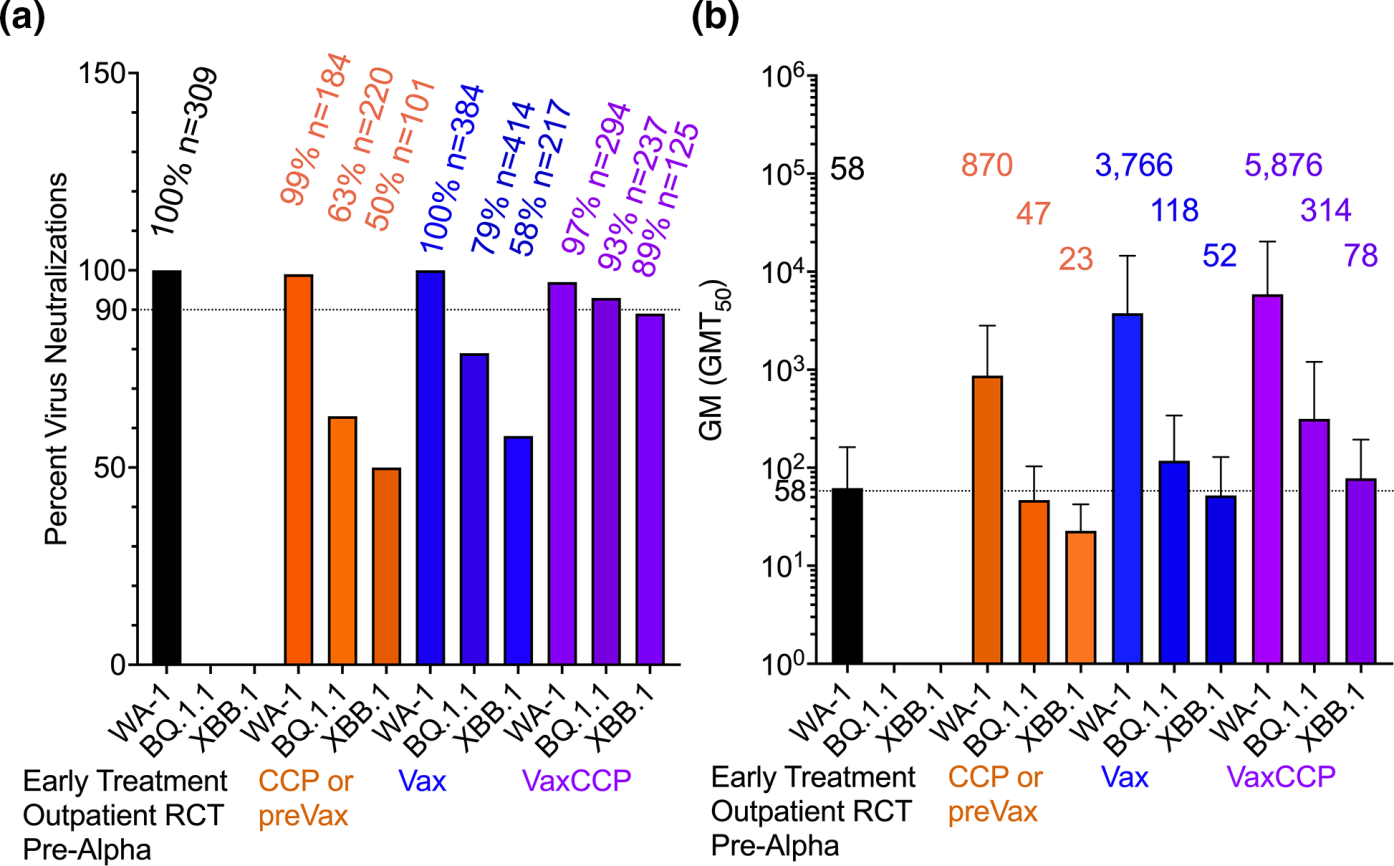
Plasma neutralizing percent and potency, (a) Total number of individual samples from cohorts in the three groups above study defined neutralization thresholds from Table 2 for WA-1, BQ.1.1 and XBB.1 graphed with pre-Alpha and Alpha CCP qualified donors (*n*=309 unique donors) effective at reducing hospitalizations in a randomized control trial treating unvaccinated outpatients. Percent virus neutralization and number in group shown above bars. (**b**) Virus neutralization GM (GMT_50_) with standard deviation from Table 2 from the three groups graphed compared to pre-Alpha and Alpha CCP qualified donors (*n*=309 unique donors). In the CCP or preVax WA-1 (*n*=10 studies), BQ.1.1 (*n*=12 studies), XBB.1 (*n*=5 studies); Vax WA-1 (*n*=19 studies), BQ.1.1 (*n*=23 studies), XBB.1 (*n*=10 studies); Vax CCP WA-1 (*n*=12 studies), BQ.1.1 (*n*=16 studies), XBB.1 (*n*=9 studies). GM (GMT50) values are above the bars.

High levels of antibodies in donor plasma from VaxCCP neutralizes more than 93 % of BQ.1.1, with XBB.1 at 89 %, only at 4–8 % drop from WA-1 (Fig. 4). Recently collected plasma in the fall of 2022 i.e.-preVax, after a 6 month window from those boosted vaccinees without prior documented COVID-19 had a 30–40 % reduction from WA-1 in neutralization percent for BQ.1.1 at 63 % and XBB.1 at 50 %. Vaccination with no documented COVID-19 fell 21 % from WA-1 with BQ.1.1 at 79 % with XBB.1 at 58 % far larger than the small less than 10 % drop in any study defined neutralizations. For historic comparison we added 309 qualified donors in the upper 60 % deciles of antibody levels of all donors with preAlpha or Alpha CCP which neutralized 100 %. When looking at the geometric mean of 58 for the 309 donors in the large randomized clinical trial effective reduction of hospitalization in outpatient COVID-19, all the GM (GMT_50_) from the studies analysed here were above except for preVax after 6 months for BQ.1.1 and Xbb.1. The GM (GMT_50_) was more than ten times higher for WA-1 neutralization. Vax GM (GMT_50_) for WA-1 was 60 times higher the early outpatient RCT with VaxCCP being 100 times higher for WA-1. Both virus neutralizations with VaxCCP for BQ.1.1 and XBB.1 were higher than the early patient RCT WA-1 neutralization with GM (GMT_50_) of 314 and 78 respectively.

Five studies used the lentiviral pseudovirus assays, with diverse Spike proteins cloned in, while the other four were live virus assays using different cell types (Table S1). Notably, Planas *et al.* employed the IGROV-1 cell type for better growth of Omicron sublineages [14]. While the single study fold reductions (FR) and percent neutralizations normalize the results between studies, the GMT_50_ can vary between studies even amongst the live authentic viral neutralization studies (e.g. mNeonGreen reporter assays versus cytopathic effects) [15, 16]. We sorted the live authentic viral neutralizations from the pseudoviral neutralizations, also plotting the minimums and maximums (Figs 1-3). In general, the live authentic SARS-CoV-2 neutralization assays for VaxCCP appeared to have similar antibody neutralization levels, with the single study by Cao *et al*. [3] employing lentiviral pseudovirus with lower dilutional titres. In contrast, the GMT_50_ achieved with pseudoviral assays in the boosted Vax without COVID-19 appeared slightly higher than the ones achieved with authentic virus.

## Discussion

The FDA deemed CCP safe and effective for both immunocompetent and IC COVID-19 outpatients [8, 9, 20], and further extended its authorized use in the IC patient population in December 2021 [9, 20], at a time when oral antiviral therapy promised a no transfusion outpatient solution and many anti-Spike mAbs were still effective.

Up until the present, CCP remained a backup bridge for IC patients, durable against the changing variants and as a salvage therapy in seronegative IC patients. With the recent advent of Omicron XBB.* and BQ.1.* defeating the remaining anti-Spike mAbs, boosted VaxCCP, recently collected within the last 6 months of either a vaccine dose or SARS-CoV-2 is likely to be the only viable remaining passive antibody therapy for IC patients who have failed to make antibodies after vaccination and still require B-cell depleting drugs or immunosuppressive therapy. In a literature review of CCP from diverse individual VOC waves (pre-Alpha, Alpha and Delta) as well as boosted vaccinees and VaxCCP up to BA-1, VaxCCP showed increasingly higher virus neutralization titres than Vax or CCP against Omicron^4^. Here we demonstrate using plasma virus neutralizations assays from the recent literature that virus neutralizations are up almost 100-fold from pre-Alpha neutralizations from an effective outpatient clinical trial (Fig. 4) in the context of 75-fold drop in recent VaxCCP from WA-1 neutralizations to XBB.1. While for some there is not a clear relationship between *in vitro* virus neutralizations and clinical outcomes for CCP, the monoclonal antibody *in vitro* virus neutralizations were used to inform clinical decision resulting in revoking authorization in the USA. While large populations are currently antibody positive from vaccinations and COVID-19, the IC populations often do not make antibody responses to either Vax or infection and these are similar to the unvaccinated seronegative population in the early pandemic for which many outpatient RCT were effective [21].

The accelerated evolution of SARS-CoV-2 VOCs has created the problem that the pharmaceutical development of additional mAbs is not worth the effort and cost given their expected short useful clinical life expectancy, so the anti-Spike mAb pipeline has remained stuck in 2022. In those vaccinated with a last dose more than 6 months prior to sample collection, both the neutralization percent and neutralizing antibody titres fell further, compared to the recently boosted VaxCCP group. Four studies (Planas [14], Zou [15], Cao [3] and Kurhade [16]) had directly comparative cohorts in the three groups which increases the robustness reduction in neutralizations with the vaccine only, or more than 6 months to last vaccine or infection event, compared to VaxCCP. The main limitation of our systematic review is the small number of studies reporting virus neutralization with BQ.1.1 with most available as pre-preprints without peer-review yet. However, we note that peer-review itself does not change GMT_50_ or neutralization numbers and the authors of these papers have considerable expertise in the topic.

Boosted VaxCCP has full potential to replace anti-Spike mAbs for passive antibody therapy of IC patients against recent Omicron sublineages, in the meanwhile polyclonal IgG formulations can be manufactured. VaxCCP qualification in the real-world will likely remain constrained on high-throughput serology, whose correlation with GMT_50_ is not perfect [22, 23]. Nevertheless, the very high prevalence (93 %) of Omicron-neutralizing antibodies and the high GM (GMT_50_) in recently boosted VaxCCP reassure about its potency, and further confirm that exact donor-recipient VOC matching is dispensable. The upper 10 % of pre-Alpha CCP neutralized the future Omicron VOC a year later in two studies [5, 24]. Overall, our findings indicate boosted VaxCCP has a diverse, robust polyclonal antibody source for virus neutralization in IC patients.

## Supplementary Data

Supplementary material 1
