# Supplementary material for: Plasma after both SARS-CoV-2 boosted vaccination and COVID-19 potently neutralizes BQ.1.1 and XBB.1
Source: J Gen Virol. 2023 May 11;104(5):001854. doi: 10.1099/jgv.0.001854 (PMC10336427; doi:10.1099/jgv.0.001854)
Supplement: Supplementary material 1 [file jgv-104-1854-s001.pdf]

Supplementary Table 1

Virus neutralization assays.

Reference	Assay	Virus	Replication-competent cells	neutralization threshold
Qu[1]	Pseudovirus	Lentiviral pseudovirus	HEK293T-ACE-2	80
Miller[2]	Pseudovirus	Lentiviral pseudovirus	HEK293T-ACE-2	20
Cao[3]	Pseudovirus	VSV pseudovirus	HEK293T-ACE-2	20
Wang[4]	Pseudovirus	VSV pseudovirus	VeroE6-	100
Ito[5]	Pseudovirus	Lentiviral pseudovirus	HEK293T-ACE-2	150
Akerman[6]	Live virus	Live authentic SARS-CoV-2	VeroE6-TMPRSS2	20
Davis[7]	Live virus	Live authentic SARS-CoV-2	VeroE6-TMPRSS2	20
Kurhade[8]	Live virus	mNeonGreen reporter USA-WA1/2020 SARS-CoV-2	Vero E6-TMPRSS2	20
Planas[9]	Live virus	Live authentic SARS-CoV-2	IGROV-1 or Vero E6-TMPRSS2	30
Zou[10]	Live virus	mNeonGreen reporter USA-WA1/2020 SARS-CoV-2	Vero E6-TMPRSS2	20

Supplementary Table 2

GMT$_{50}$ of different plasma sources against BQ.1.1, BA.4/5, BA.4.6, BA.2.75, XBB.1 and BF.7 and fold-reductions (FR) compared to WA-1.

papers	Vaccine and COVID-19 history at sample time	Group	#	WA-1 GMT$_{50}$	BQ.1.1 GMT$_{50}$	FR BQ.1.1	BA.4/5 GMT$_{50}$	FR BA.4/5	BA.4.6 GMT$_{50}$	FR BA.4.6	BA.2.75 GMT$_{50}$	FR BA.2.75	XBB.1 GMT$_{50}$	FR XBB	BF.7 GMT$_{50}$	FR BF.7
Cao[3]	3xCorVac+BA.1 BTI	VaxCCP	50	1557	27	58	107	15	23	68	197	8	23	68	70	22
Cao[3]	3xCorVac +BA.2 BTI	VaxCCP	39	1245	40	31	175	7	22	57	217	6	23	54	97	13
Cao[3]	3xCorVac +BA.5 BTI	VaxCCP	36	1136	77	15	508	2	27	42	145	8	27	42	208	5
Zou[10]	4xBNT162b2+BTI	VaxCCP	20	5120	132	39	629	8	587	9	265	19	99	52		
Planas[9]	3xmRNAvac+ BA.1/2 BTI	VaxCCP	13	8000	200	40	400	20	500	16	200	40				
Wang[4]	2-3xmRNAvac+ BA.2 BTI	VaxCCP	14	24970	849	29	3727	7					186	134		
Wang[4]	2-3xmRNAvac+ BA.2 BTI--BQ.1	VaxCCP	14		1250	20										
Wang[4]	3-4xmRNAvac+ BA.4/5 BTI	VaxCCP	20	20507	671	31	5541	4					214	96		
Wang[4]	3-4xmRNAvac+ BA.4/5 BTI--BQ.1	VaxCCP	20		1644	12										
Kurhade[8]	3xmRNAvac+bivalent+ BTI	VaxCCP	23	5776	267	22	1558	4	744	8	367	16	103	56	1223	5
Ito[5]	2-3xmRNAvac+BA.2 BTI	VaxCCP	14		400	7	800									
Ito[5]	2-3xmRNAvac+BA.5 BTI	VaxCCP	20		400	5	2100									
Zou[10]	3xBNT162b2+bivalent+ BTI	VaxCCP	19	4847	444	11	1377	4	1564	3	326	15	131	37		
Akerman[6]	3xmRNA +bivalent	VaxCCP	29	1748	88	20	468	4	289	6			139	13		
Planas[9]	3xmRNAvac+ BA.1/2 BTI	VaxCCP	16	25000	700	36	1000	25	2000	13	600	42				
Planas[9]	3xmRNAvac+ BA.5 BTI	VaxCCP	15	30000	3000	10	10000	3	9000	3	900	33				
Davis[7]	3xmRNAvac	Vax	12	758	19	40	50	15			23	33				
Kurhade[8]	4xmRNAvac	Vax	25	1533	22	70	95	16	62	25	26	59	15	102	69	22
Cao[3]	3xCorVac	Vax	40	652	24	27	72	9	21	31	90	7	20	33	45	14
Zou[10]	4xBNT162b2	Vax	20	1325	26	51	89	15	92	14	37	36	17	78		
Planas[9]	3xmRNAvac	Vax	10	1500	40	38	60	25	60	25	10	150				
Wang[4]	3xmRNAvac	Vax	14	7687	139	55	628	12					108	71		
Wang[4]	3xmRNAvac--BQ.1	Vax	14		208	37										

Wang[4]	3xmRNAvac+monovalent	Vax	19	21182	261	81	1540	14					137	155		
Wang[4]	3xmRNAvac+monovalent--BQ.1	Vax	19		496	43										
Wang[4]	3xmRNAvac+bivalent	Vax	21	13736	337	41	1688	8					162	85		
Wang[4]	3xmRNAvac+bivalent--BQ.1	Vax	21		568	24										
Davis[7]	3xmRNAvac+monovalent	Vax	12	1812	53	34	142	13			65	28				
Akerman[6]	4xmRNA	Vax	23	1141	80	14	257	4	179	6			117	10		
Akerman[6]	3xmRNAvac after 2020 WA-1	Vax	47	1075	70	15	212	5	110	10			54	20		
Kurhade[8]	3xmRNAvac+bivalent	Vax	29	3620	73	50	298	12	183	20	98	37	35	103	305	12
Davis[7]	3xmRNAvac+bivalent	Vax	12	2312	112	21	576	4			201	12				
Qu[1]	3xmRNAvac--BQ.1	Vax	15		140	19										
Qu[1]	3xmRNAvac	Vax	15	2616	114	23	300	9	246	11	589	4			238	11
Zou[10]	3xBNT162b2+bivalent	Vax	18	2237	143	16	518	4	524	4	117	19	55	41		
Planas[9]	3xmRNAvac	Vax	18	6000	200	30	300	20	300	20	60	100				
Miller[2]	3xBNT162b2	Vax	16	45695	261	175	887	52			387	118			595	77
Miller[2]	3xmRNA+ monovalent	Vax	18	21507	406	53	2829	8			745	29			2276	9
Miller[2]	3xmRNA +bivalent	Vax	15	40515	508	80	3693	11			883	46			2399	17
Qu[1]	BA.4/5 inf (17-unvac)	CCP	20	707	66	11	190	4	180	4	210	3			162	4
Qu[1]	BA.4/5 inf (17-unvac)--BQ.1	CCP	20		68	10										
Qu[1]	Hosp BA.1 (6-unvac;5-2xmRNAvac)	CCP	15	720	145	5	263	3	205	4	186	4			227	3
Qu[1]	Hosp BA.1 (6-unvac;5-2xmRNAvac)--BQ.1	CCP	15		135	5										
Zou[10]	3xBNT162b2+BTI	PreVax with BNT162b	20	2516	60	42	226	11	283	9	126	20	55	46		
Zou[10]	3xBNT162b2 +BTI	PreVax with bivalent	19	1377	74	19	207	7	282	5	62	22	27	51		
Zou[10]	3xBNT162b2	PreVax with bivalent	18	**226**	**11**	**21**	**20**	**11**	**24**	**9**	**14**	**16**	**12**	**19**		
Zou[10]	3xBNT162b2	PreVax with BNT162b	20	**303**	**17**	**18**	**30**	**10**	**36**	**8**	**18**	**17**	**13**	**23**		
Akerman[6]	3xmRNA	PreVax	47	**203**	**34**	**6**	**63**	**3**	**50**	**4**			**26**	**8**		
Miller[2]	2xBNT162b2	PreVax with BNT162b	16	**484**	**20**	**24**	**20**	**24**			**20**	**24**			**20**	**24**
Miller[2]	3xmRNA	PreVax with bivalent	15	**3633**	**45**	**81**	**211**	**17**			**33**	**110**			**131**	**28**
Miller[2]	3xmRNA	PreVax with monovalent	18	**5731**	**49**	**117**	**184**	**31**			**117**	**49**			**168**	**34**

Supplementary Table 3

Neutralizing activity numbers (#) by study cohort.

papers	Vaccine and COVID-19 history at sample time	groups	WA-1 number	WA-1 neutralizing number	BQ.1.1 number	BQ.1.1 neutralizing number	BA.4/5 number	BA.4/5 neutralizing number	BA.4.6 number	BA.4.6 neutralizing number	BA.2.75 number	BA.2.75 neutralizing number	XBB.1 number	XBB.1 neutralizing number	BF.7 number	BF.7 neutralizing number
Cao[3]	3xCorVac+BA.1 BTI	VaxCCP	50	50			50	50			50	50			50	49
Cao[3]	3xCorVac +BA.2 BTI	VaxCCP	39	39			39	39			39	39			39	38
Cao[3]	3xCorVac +BA.5 BTI	VaxCCP	36	36			36	36			36	36			36	36
Zou[10]	4xBNT162b2+BTI	VaxCCP	20	20	20	20	20	20	20	20	20	20	20	19		
Planas[9]	3xmRNAvac+ BA.1/2 BTI	VaxCCP	13	13	13	11	13	12	13	13	13	11				
Wang[4]	2-3xmRNAvac+ BA.2 BTI	VaxCCP	14	14	14	13	14	14					14	8		
Wang[4]	2-3xmRNAvac+ BA.2 BTI--BQ.1	VaxCCP			14	13										
Wang[4]	3-4xmRNAvac+ BA.4/5 BTI	VaxCCP	20	20	20	18	20	20					20	14		
Wang[4]	3-4xmRNAvac+ BA.4/5 BTI--BQ.1	VaxCCP			20	20										
Kurhade [8]	3xmRNAvac+bivalent+BTI	VaxCCP	23	23	23	22	23	23	23	23	23	23	23	22	23	23
Ito[5]	2-3xmRNAvac+BA.2 BTI	VaxCCP			14	11	14	13								
Ito[5]	2-3xmRNAvac+BA.5 BTI	VaxCCP			20	14	20	20								
Zou[10]	3xBNT162b2+bivalent+BTI	VaxCCP	19	19	19	19	19	19	19	19	19	20	19	19		
Akerman[6]	3xmRNA +bivalent	VaxCCP	29	20	29	29	29	29	29	29			29	29		
Planas[9]	3xmRNAvac+ BA.1/2 BTI	VaxCCP	16	16	16	16	16	16	16	16	16	16				
Planas[9]	3xmRNAvac+ BA.5 BTI	VaxCCP	15	15	15	15	15	15	15	15	15	15				
Davis[7]	3xmRNAvac	Vax	12	12	12	6	12	11			12	9				
Kurhade [8]	4xmRNAvac	Vax	25	25	25	15	25	23	25	22	25	17	25	8	25	21
Cao[3]	3xCorVac	Vax	40	40			40	39			40	40			40	37
Zou[10]	4xBNT162b2	Vax	20	20	20	15	20	8	20	19	20	19	20	10		

Planas[9]	3xmRNAvac	Vax	10	10	10	6	10	7	10	5	10	3				
Wang[4]	3xmRNAvac	Vax	14	14	14	5	14	14					14	3		
Wang[4]	3xmRNAvac--BQ.1	Vax			14	6										
Wang[4]	3xmRNAvac+monovalent	Vax	19	19	19	15	19	19					19	8		
Wang[4]	3xmRNAvac+monovalent--BQ.1	Vax			19	16										
Wang[4]	3xmRNAvac+bivalent	Vax	21	21	21	16	21	21					21	9		
Wang[4]	3xmRNAvac+bivalent--BQ.1	Vax			21	18										
Davis[7]	3xmRNAvac+monovalent	Vax	12	12	12	9	12	12			12	10				
Akerman[6]	4xmRNA	Vax	23	23	23	20	23	23	23	22			23	17		
Akerman[6]	3xmRNAvac after 2020 WA-1	Vax	47	46	47	34	47	45	47	43			47	32		
Kurhade [8]	3xmRNAvac+bivalent	Vax	29	29	29	26	29	29	29	28	29	28	29	20	29	28
Davis[7]	3xmRNAvac+bivalent	Vax	12	12	12	10	12	12			12	11				
Qu[1]	3xmRNAvac--BQ.1	Vax			15	14										
Qu[1]	3xmRNAvac	Vax	15	15	15	12	15	15	15	15	15	14			15	14
Zou[10]	3xBNT162b2+bivalent	Vax	18	18	19	18	18	18	19	19	19	18	19	18		
Planas[9]	3xmRNAvac	Vax	18	18	18	16	18	18	18	18	18	13				
Miller[2]	3xBNT162b2	Vax	16	16	16	16	16	16			16	16			16	16
Miller[2]	3xmRNA+ monovalent	Vax	18	18	18	18	18	18			18	18			18	18
Miller[2]	3xmRNA +bivalent	Vax	15	15	15	15	15	15			15	15			15	15
Qu[1]	BA.4/5 inf (17-unvac)	CCP	20	20	20	12	20	15	20	14	20	15			20	14
Qu[1]	BA.4/5 inf (17-unvac)--BQ.1	CCP			20	11										
Qu[1]	Hosp BA.1 (6-unvac;5-2xmRNAvac)	CCP	15	14	15	13	15	11	15	12	15	8			15	14
Qu[1]	Hosp BA.1 (6-unvac;5-2xmRNAvac)--BQ.1	CCP			15	14										
Zou[10]	3xBNT162b2+BTI	PreVax with BNT162b	20	20	20	16	20	19	20	19	20	17	20	17		

Zou[10]	3xBNT162b2 +BTI	PreVax with bivalent	19	19	19	16	19	19	19	19	19	17	19	12		
Zou[10]	3xBNT162b2	PreVax with bivalent	18	18	19	1	18	9	19	9	19	5	19	2		
Zou[10]	3xBNT162b2	PreVax with BNT162 b	20	19	20	6	20	12	20	12	20	8	20	5		
Akerma n[6]	3xmRNA	PreVax	23	23	23	16	23	21	23	19			23	14		
Miller[2]	2xBNT162b2	PreVax with BNT162 b	16	16	16	6	16	5			16	7			16	4
Miller[2]	3xmRNA	PreVax with bivalent	15	15	15	13	15	15			15	9			15	14
Miller[2]	3xmRNA	PreVax with monoval ent	18	18	18	15	18	18			18	18			18	17

Supplementary Figure 1

Plasma GMT_{50} from post boosted vaccinations and COVID-19 (VaxCCP) sorted by study cohort with live virus assays on the left and pseudovirus on right, with individual sample minimum and maximum dilution titer also shown. A) WA-1; B) BQ.1.1; C) BA.2.75; D) BA.4/5.

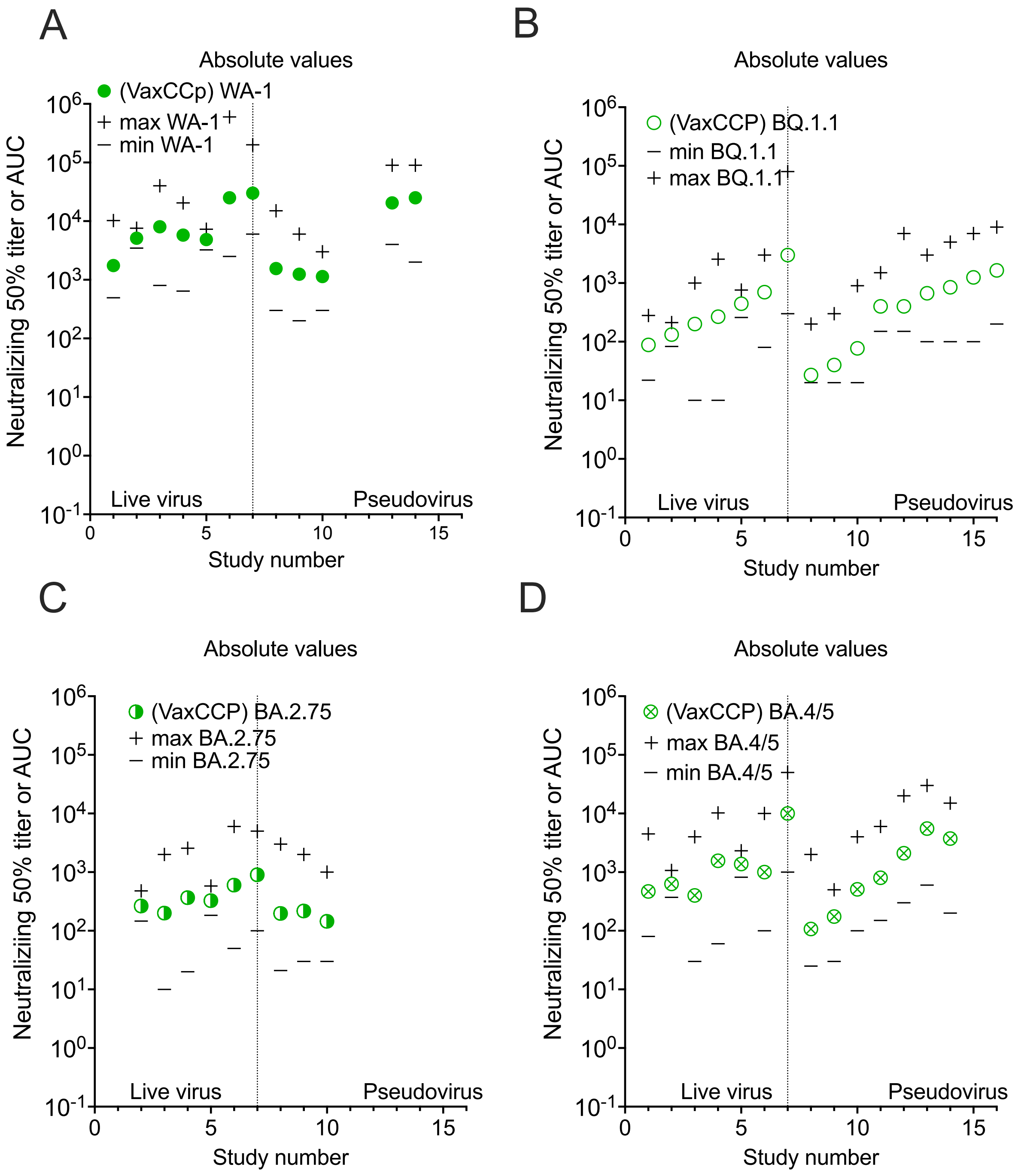

Supplementary Figure 2

Plasma GMT$_{50}$ from boosted vaccinations (Vax) only without COVID-19 sorted by study cohort with live virus assays on the left and pseudovirus on right with with individual sample minimum and maximum dilution titer also shown. A) WA-1; B) BQ.1.1; C) BA.2.75; and D) BA.4/5.

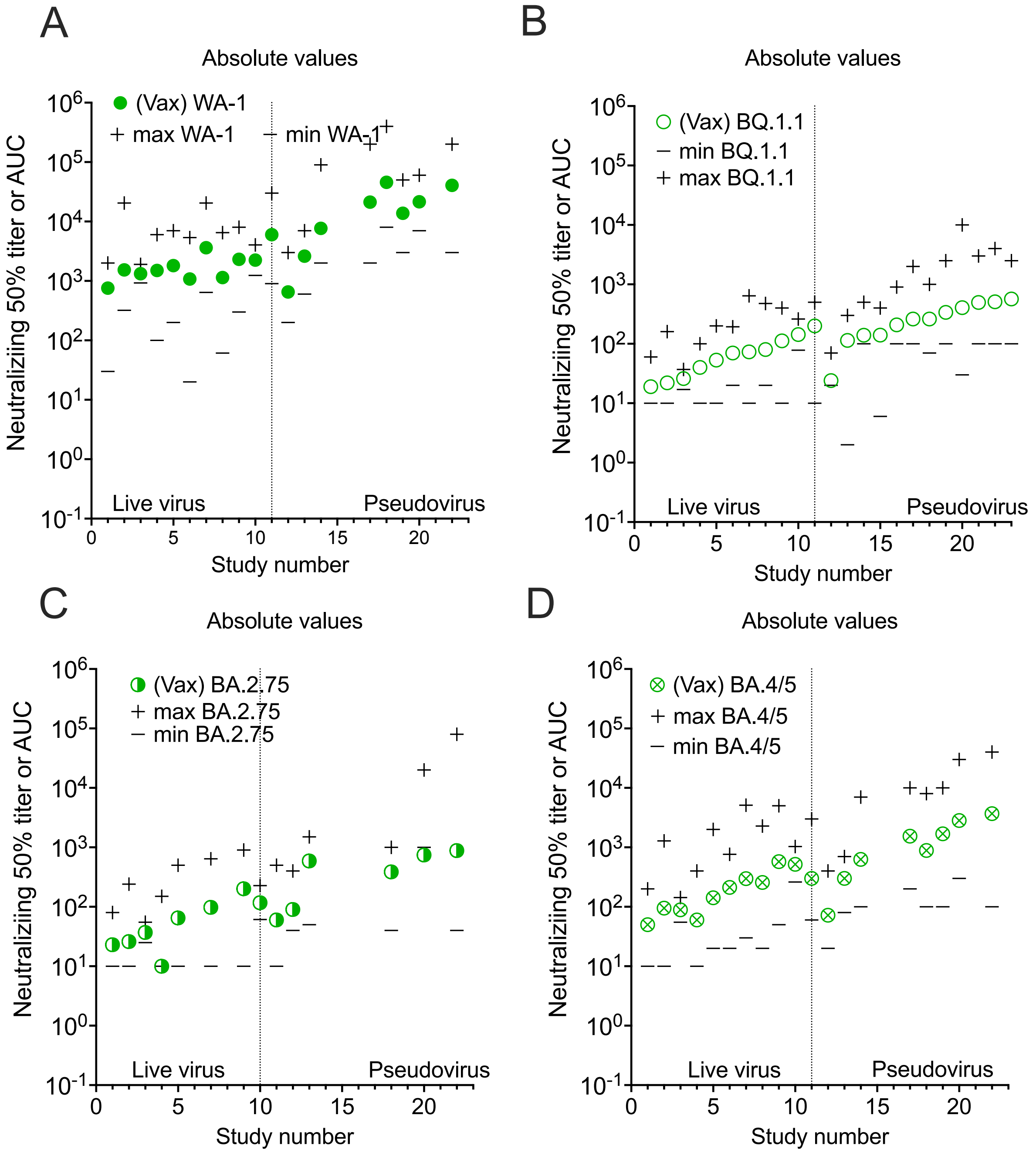

Supplementary Figure 3

Plasma GMT_{50} from Omicron infection alone (CCP) and also pre-boosted vaccination (preVax) in 2021 or 2022 6 to 11 months after last vaccine dose sampled sorted by study cohort with live virus assays on the left and pseudovirus on right with minimum and maximum dilution titer also shown. A) WA-1; B) BQ.1.1; C) BA.2.75; and D) BA.4/5.

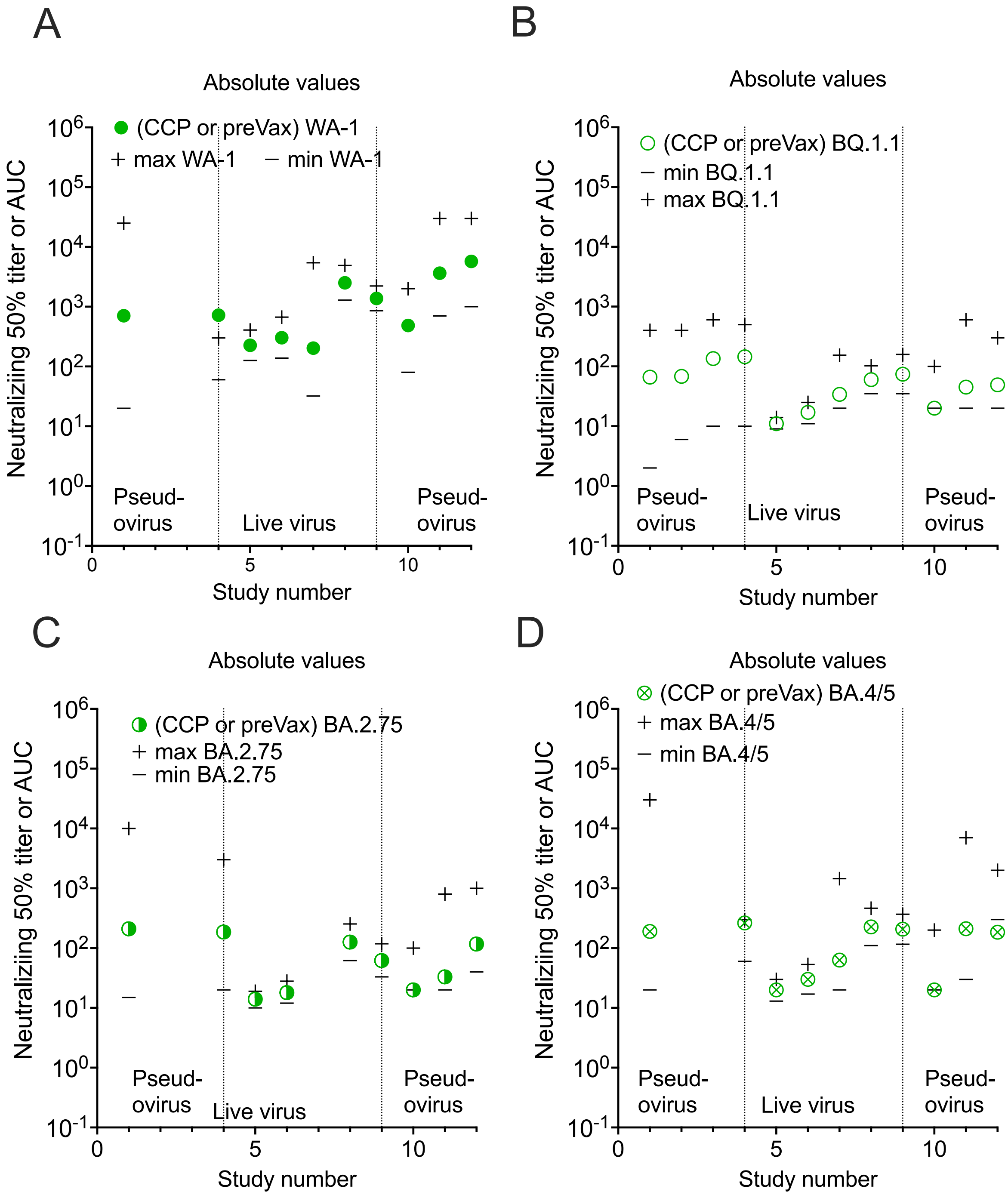

References

1. **Qu P, Evans JP, Faraone J, Zheng Y-M, Carlin C et al.** Distinct Neutralizing Antibody Escape of SARS-CoV-2 Omicron Subvariants BQ.1, BQ.1.1, BA.4.6, BF.7 and BA.2.75.2. *bioRxiv* 2022:2022.2010.2019.512891.

2. **Miller J, Hachmann NP, Collier A-rY, Lasrado N, Mazurek CR et al.** Substantial Neutralization Escape by the SARS-CoV-2 Omicron Variant BQ.1.1. *bioRxiv* 2022:2022.2011.2001.514722.

3. **Cao Y, Jian F, Wang J, Yu Y, Song W et al.** Imprinted SARS-CoV-2 humoral immunity induces convergent Omicron RBD evolution. *bioRxiv* 2022:2022.2009.2015.507787.

4. **Wang Q, Iketani S, Li Z, Liu L, Guo Y et al.** Alarming antibody evasion properties of rising SARS-CoV-2 BQ and XBB subvariants. *bioRxiv* 2022:2022.2011.2023.517532.

5. **Ito J, Suzuki R, Uriu K, Itakura Y, Zahradnik J et al.** Convergent evolution of the SARS-CoV-2 Omicron subvariants leading to the emergence of BQ.1.1 variant. *bioRxiv* 2022:2022.2012.2005.519085.

6. **Akerman A, Milogiannakis V, Jean T, Esneu C, Silva MR et al.** Emergence and antibody evasion of BQ and BA.2.75 SARS-CoV-2 sublineages in the face of maturing antibody breadth at the population level. *medRxiv* 2022:2022.2012.2006.22283000.

7. **Davis-Gardner ME, Lai L, Wali B, Samaha H, Solis D et al.** mRNA bivalent booster enhances neutralization against BA.2.75.2 and BQ.1.1. *bioRxiv* 2022:2022.2010.2031.514636.

8. **Kurhade C, Zou J, Xia H, Liu M, Chang HC et al.** Low neutralization of SARS-CoV-2 Omicron BA.2.75.2, BQ.1.1, and XBB.1 by 4 doses of parental mRNA vaccine or a BA.5-bivalent booster. *bioRxiv* 2022:2022.2010.2031.514580.

9. **Planas D, Bruel T, Staropoli I, Guivel-Benhassine F, Porrot F et al.** Resistance of Omicron subvariants BA.2.75.2, BA.4.6 and BQ.1.1 to neutralizing antibodies. *bioRxiv* 2022:2022.2011.2017.516888.

10. **Zou J, Kurhade C, Patel S, Kitchin N, Tompkins K et al.** Improved Neutralization of Omicron BA.4/5, BA.4.6, BA.2.75.2, BQ.1.1, and XBB.1 with Bivalent BA.4/5 Vaccine. *bioRxiv* 2022:2022.2011.2017.516898.
